# Randomised phase 3 open-label trial of first-line treatment with gemcitabine in association with docetaxel or paclitaxel in women with metastatic breast cancer: a comparison of different schedules and treatments

**DOI:** 10.1186/1471-2407-13-164

**Published:** 2013-03-28

**Authors:** Lucia Del Mastro, Alessandra Fabi, Mauro Mansutti, Michele De Laurentiis, Antonio Durando, Domenico Franco Merlo, Paolo Bruzzi, Ignazia La Torre, Matteo Ceccarelli, Gbenga Kazeem, Paolo Marchi, Davide Boy, Marco Venturini, Sabino De Placido, Francesco Cognetti

**Affiliations:** 1 IRCCS AOU San Martino - IST - National Institute for Cancer Research, UO Sviluppo Terapie Innovative, Genoa, Italy; 2 Division Oncology, Regina Elena Institute, Rome, Italy; 3 Department Oncology, S. Maria della Misericordia Hospital, Udine, Italy; 4 Department of Endocrinology and Molecular and Clinical Oncology, University of Naples Federico II, Naples, Italy; 5 Department of Senology, Division of Breast Oncology, National Cancer Institute “Fondazione Pascale”, Naples, Italy; 6 A.O.O.I. Regina Margherita S. Anna, Turin, Italy; 7 IRCCS AOU San Martino - IST - National Institute for Cancer Research, UO Epidemiologia Clinica, Genoa, Italy; 8 Eli Lilly Italy, Medical Department, Sesto Fiorentino, FI, Italy; 9 Eli Lilly UK, Erl Wood, Surrey, UK; 10 Division Oncology, S. Cuore – Don Calabria Hospital, Verona, Italy

**Keywords:** Metastatic breast cancer, Weekly schedule, 3-weekly schedule

## Abstract

**Background:**

This open-label study compared docetaxel/gemcitabine vs. paclitaxel/gemcitabine and a weekly (W) vs. 3-weekly (3 W) schedule in metastatic breast cancer (MBC).

**Methods:**

Patients relapsed after adjuvant/neoadjuvant anthracycline-containing chemotherapy were randomized to: A) gemcitabine 1000 mg/m^2^ Day 1,8 + docetaxel 75 mg/m^2^ Day 1 q3W; B) gemcitabine 1250 mg/m^2^ Day 1,8 + paclitaxel 175 mg/m^2^ Day 1 q3W; C) gemcitabine 800 mg/m^2^ Day 1,8,15 + docetaxel 30 mg/m^2^ Day 1,8,15 q4W; D) gemcitabine 800 mg/m^2^ Day 1,15 + paclitaxel 80 mg/m^2^ Day 1,8,15 q4W. Primary endpoint was time-to-progression (TTP). Secondary endpoints were overall survival (OS) and overall response rate (ORR).

**Results:**

Interim analysis led to accrual interruption (241 patients enrolled of 360 planned). Median TTP (months) was 8.33 (95% CI: 6.19-10.16) with W and 7.51 (95% CI: 5.93-8.33) with 3 W (p=0.319). No differences were observed in median TTP between docetaxel and paclitaxel, with 85.6% and 87.0% of patients progressing, respectively. OS did not differ between regimens/schedules. ORR was comparable between regimens (HR: 0.882; 95% CI: 0.523-1.488; p=0.639), while it was significantly higher in W than in the 3 W (HR: 0.504; 95% CI: 0.299-0.850; p=0.010) schedule. Grade 3/4 toxicities occurred in 69.2% and 71.9% of patients on docetaxel and paclitaxel, and in 65.8% and 75.2% in W and 3 W.

**Conclusions:**

Both treatment regimens showed similar TTP. W might be associated with a better tumour response compared with 3 W.

**Trial registration:**

Clinicaltrial.gov ID NCT00236899

## Background

Approximately 4%-6% of breast cancer is metastatic at diagnosis and, depending on prognostic factors, up to 30% of node negative and 70% of node positive breast cancer will relapse [1]. Although the vast majority of metastatic breast cancer cases are incurable, the main goal of treatment is not only palliation but also survival improvement. Chemotherapy is the cornerstone of therapy for patients not candidates for endocrine therapy. The widespread inclusion of anthracyclines in the adjuvant setting limits their use as first-line therapy in metastatic disease. Cytotoxic drugs with activity in advanced breast cancer include the taxanes (paclitaxel and docetaxel), gemcitabine, vinorelbine and capecitabine. The doublet of paclitaxel and gemcitabine is superior in terms of overall survival to monotherapy with paclitaxel [2]. Also the combination of docetaxel and capecitabine is superior to monotherapy but produced significant toxicity [3]. The regimen of gemcitabine plus docetaxel showed similar activity but less toxicity compared to capecitabine plus docetaxel [4]. No direct comparisons between these two doublets (gemcitabine plus paclitaxel and gemcitabine plus docetaxel) are available so far.

Both paclitaxel and docetaxel can be administered as weekly or three-weekly regimens and, at the time of the study design, the most suitable regimen was still unknown.

Based on this background, the present Phase III trial was designed to compare the two doublets docetaxel/gemcitabine and paclitaxel/gemcitabine in terms of efficacy and safety, as well as the use of a weekly schedule over a standard 3-weekly regimen.

## Methods

### Patients

The study population included adult women with Human Epidermal Growth Factor Receptor 2 (HER-2) negative MBC who relapsed after receiving one adjuvant/neoadjuvant chemotherapy treatment containing an anthracycline, unless clinically contraindicated. Table 1 summarizes the main inclusion and exclusion criteria for entry in the study.

**Table 1:** Main inclusion and exclusion criteria for entry in the study

**Inclusion criteria**	**Exclusion criteria**
Adult women with HER-2 negative MBC	Previous chemotherapy for metastatic disease
MBC relapsed after receiving one adjuvant/neoadjuvant chemotherapy containing an anthracycline, unless clinically contraindicated	Previous chemotherapy with gemcitabine in any setting of disease
Could have received a prior neoadjuvant or adjuvant taxanes regimen as long as it was ≥12 months since completion of the treatment	Patients with second primary malignancy (except in situ carcinoma of the cervix or adequately treated non-melanoma carcinoma of the skin or other malignancy treated at least 5 years previously with no evidence of recurrence)
Measurable disease as defined by RECIST 1.0 (however patients with only bone metastases were included in the study),	Pre-existing sensorial or motor neuropathy NCI-CTC grade >1
Previous hormonal therapy for adjuvant setting or metastatic disease (≤2 lines), or immunotherapy, was allowed and should have been completed before the enrolment	Inflammatory breast cancer without evidence of metastatic disease
Performance status ≥70 on the Karnofsky Scale	Patients with serious concomitant systemic disorders (e.g., uncontrolled active cardiovascular diseases and/or myocardial infarction within the preceding 6 months)
Life expectancy ≥12 weeks	Patients with clinical evidence of symptomatic brain metastasis
Adequate bone marrow and liver/renal function	Pregnancy or breast-feeding
Prior radiotherapy should have been completed 4 weeks before study entry	Patients with reproductive potential not using an approved contraceptive method if appropriate (except hormonal substitutive therapy)

HER-2: Human Epidermal Growth Factor Receptor 2, MBC: Metastatic Breast Cancer, NCI-CTC: National Cancer Institute-Common Toxicity Criteria, RECIST: Response Evaluation Criteria in Solid Tumours.

The participant patients gave their written informed consent prior to entering the study. The study protocol and the informed consent forms were reviewed and approved by the Independent Ethics Committees of each participating centre before any study-related procedure was started.

### Study design and treatments

This was a multi-centre, open-label, 2x2 factorial randomised study (clinicaltrial.gov ID: http://NCT00236899) in which eligible patients were equally randomised (using random-number table with “investigational centre” as stratification factor) to one of the following four treatment arms: Arm A: gemcitabine 1000 mg/m^2^ administered intravenously (IV) on Days 1 and 8 + docetaxel 75 mg/m^2^ IV on Day 1 of each 21-day cycle (3-weekly); Arm B: gemcitabine 1250 mg/m^2^ IV on Days 1 and 8 + paclitaxel 175 mg/m^2^ IV on Day 1 of each 21-day cycle (3-weekly); Arm C: gemcitabine 800 mg/m^2^ IV on Days 1, 8 and 15 + docetaxel 30 mg/m^2^ IV on Days 1, 8 and 15 of each 28-day cycle (weekly); Arm D: gemcitabine 800 mg/m^2^ IV on Days 1, 8 and 15 + paclitaxel 80 mg/m^2^ on Days 1, 8 and 15 of each 28-day cycle (weekly).

A maximum of 6 cycles was scheduled in case of stable disease (SD, defined as neither sufficient shrinkage to qualify for partial response nor suffiecient increase to qualify for progression taking as reference the smallest sum of the longest diameters since the treatment started), to be continued up to 10 cycles for observed partial or complete response, respectively. Patients were to be discontinued from the study in the case of evidence of progressive disease or unacceptable toxicity.

Dose adjustments were made based on the worst NCI-common toxicity criteria (NCI-CTC version 3.0), toxicity experienced by the patient in the previous cycle. Any patient with two prior dose reductions who experienced a toxicity that would cause a third dose reduction had to be discontinued from study therapy.

No other chemotherapy, biological therapy, immunotherapy (such as trastuzumab), hormonal therapy (excluding corticosteroids) or experimental medications were permitted while patients were on the study. Bisphosphonate therapy was allowed at the discretion of the investigator. Palliative radiation on painful lesions was allowed, provided that at least 2 weeks elapsed between the use of gemcitabine and radiotherapy. Patients could receive growth factors for hematologic toxicity, prophylactic antiemetics and/or premedication agents (e.g., corticosteroids).

### Outcome measures

Tumour outcomes were evaluated every two cycles according to RECIST 1.0 criteria. Time-to-progression (TTP) was defined as the time from the first day of treatment to first observation of documented disease progression or death due to any cause. TTP was censored at the time of last follow-up for those patients who were still alive without progression. Other efficacy measures were overall survival (OS), defined as time from enrolment to time of death as a result of any cause (for patients still alive, OS was censored at the last contact). Overall response rate (ORR) was evaluated every two cycles according to RECIST 1.0 criteria. Best overall response was the best response recorded from the start of treatment until disease progression; complete and partial responses (CR/PR) were to be confirmed by two evaluations of the disease, taken at least 4 weeks apart; stable disease (SD) was accepted if one measurement was provided at least 9 weeks from baseline.

Safety analyses included summary of adverse event rates and laboratory changes, summary of the number of the NCI-CTC (version 3.0) toxicities grade for laboratory and non-laboratory parameters. Toxicity was evaluated on Day 1 of every cycle and at 30 days post study. On-study evaluation of haematology occurred on Days 1 and 8 in Arms A and B, and on Days 1, 8 and 15 of every cycle in arms C and D. Post therapy assessment of haematology occurred not earlier than 30 days after completion of the last treatment cycle.

Quality of Life (QoL) was measured on Day 1 of every cycle and at 30 days post study, using the Rotterdam Symptom Checklist (RSCL) [5]. The RSCL was assessed for each patient no more than one week before entering the study, every 3 to 4 weeks and at 30 days post therapy visit.

### Statistical considerations

The primary objectives of the study were 1) to compare TTP in patients with metastatic breast cancer (MBC) treated with gemcitabine plus docetaxel to patients treated with gemcitabine plus paclitaxel and 2) to compare TTP in MBC patients treated with a weekly schedule to patients treated with the standard three-weekly schedule. The planned sample size of 360 patients was chosen to allow the observation of 252 events, which gives 80% power of rejecting the null hypothesis of no difference in TTP rates against an alternative hypothesis of a 30% reduction in TTP rates between the treatment groups (schedules or drugs) assuming a two-sided significance level of 5%. These assumptions were based on a constant rate of accrual of 120 patients per year over a 3-year period.

Analyses for TTP, OS and QoL were conducted on all randomised patients according to the intent-to-treat (ITT) principle. For tumour response rates and safety, analyses were performed for all randomised patients who received at least one dose of docetaxel, paclitaxel or gemcitabine.

For each of the time-to-event endpoints, Kaplan-Meier curves were generated, and quartiles and point probabilities were calculated. Interval estimates were obtained using 95% CIs.

A multivariate Cox proportional hazard analysis that used covariates that could influence the time-to-event endpoints (i.e., presence/absence of visceral metastases, menopausal status, prior adjuvant/neoadjuvant taxane therapy, prior hormonal therapy, treatment schedule, treatment drug) was performed for TTP and OS. Response rate and 95% CI were calculated. In the analysis of ORR, a multivariate logistic regression analysis, which included the same covariates as those of the Cox’s model, was also performed. For the QoL measures, descriptive statistics (number of response and percentages) were tabulated for each treatment arm.

Safety analyses included summaries of the blood/platelet transfusion required, summary of adverse events rates and laboratory changes, summary of the number of the NCI-CTC (version 3.0) toxicities grade for laboratory and non-laboratory parameters.

### Interim futility analysis

The slow rate of accrual in the trial prevented the completion of the planned patients’ enrolment within a reasonable time. Consequently, an interim futility analysis was performed to evaluate if the study should be stopped early for futility. The interim futility analysis was based on all events (progressions or deaths without documented progression) that occurred before 30 November 2008 (i.e., more than 3 years from the start of the trial). With 100-110 events, the expectation was as follows: i) If HR >1 was obtained, then there was a strong support for stopping patients’ accrual for futility; ii) If HR <0.85 was observed, then the accrual should have continued as planned.

## Results

### Interim futility analysis

Two hundred and fifty-two patients entered the futility analysis and 113 events (56%) were observed. This constituted about 45% (113/252) of the planned number of events. The HR for TTP comparisons was 1.06 (95% CI: 0.73-1.54) for the docetaxel arm versus paclitaxel treatment arm and 1.04 (95% CI: 0.72-1.51) for the weekly versus 3-weekly schedules comparison. Based on these results, it was estimated that if the original alternative hypothesis (HR= 0.7) was true and the study was brought to its natural conclusion, the chance (that is, conditional power) of observing a significant difference in favour of the docetaxel arm was 16% and only 6% between the treatment schedules. On the basis of the results of the futility analyses, the two alternative hypotheses for the primary endpoint were less likely than when the study was initiated and, consequently, it was considered as not appropriate to continue the patient accrual for the study.

### Patient disposition and treatment compliance

**Figure 1:**
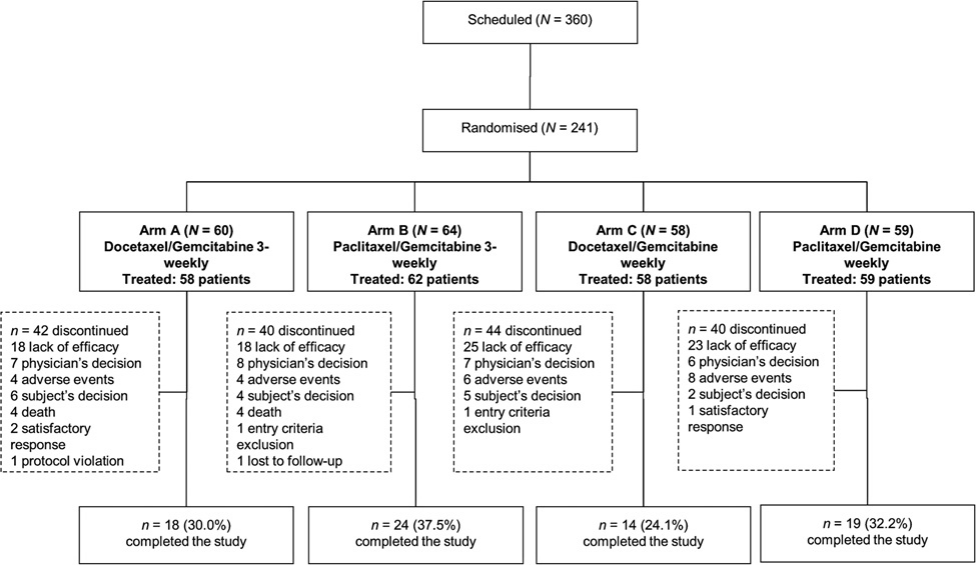
Patients disposition and reasons for study discontinuation.

Overall, 241 patients were enrolled between September 2005 and August 2010 and were randomised as follow: 60 patients (24.9%) in Arm A (3-weekly docetaxel/gemcitabine), 64 (26.6%) in Arm B (3-weekly paclitaxel/gemcitabine), 58 (24.1%) in Arm C (weekly docetaxel/gemcitabine) and 59 (24.5%) in Arm D (weekly paclitaxel/gemcitabine). Treatment arms were balanced in accordance with the baseline factor “investigational centre”. Patient disposition and reasons for discontinuation are reported in Figure 1.

Table 2 shows the demographic and other baseline characteristics of the study population. There were no substantial differences between groups for age, menopausal status, hormonal receptor status, use of previous hormonal therapy and presence of visceral metastases, while fewer patients in Arm C than in the other three groups had received previous adjuvant/neoadjuvant taxane therapy.

**Table 2:** Demographic and other baseline characteristics

		**Arm A**	**Arm B**	**Arm C**	**Arm D**	**Overall**
		**(*N*=60)**	**(*N*=64)**	**(*N*=58)**	**(*N*=59)**	**(*N*=241)**
**Age (years)**						
Median (Range)		58.5 (37-76)	57.5 (31-74)	56 (38-77)	55 (33-76)	57 (31-77)
		*N* (%)	*N* (%)	*N* (%)	*N* (%)	*N* (%)
Ethnic Origin, N (%)	Caucasian	58 (96.6)	63 (98.4)	58 (100)	59 (100)	238 (98.8)
	African	1 (1.7)	0 (0.0)	0 (0.0)	0 (0.0)	1 (0.4)
	Asian	1 (1.7)	0 (0.0)	0 (0.0)	0 (0.0)	1 (0.4)
	Other^a^	0 (0.0)	1 (1.6)	0 (0.0)	0 (0.0)	1 (0.4)
Menopausal status, N (%)	pre	19 (31.7)	17 (26.6)	16 (27.6)	21 (35.6)	73 (30.3)
	post	41 (68.3)	47 (73.4)	42 (72.4)	38 (64.4)	168 (69.7)
**Karnofsky Performance Status, N (%)**	Point					
Able to care for self	70	0 (0.0)	0 (0.0)	2 (3.5)	2 (3.4)	4 (1.66)
Normal activity but requiring effort	80	5 (8.3)	3 (4.7)	5 (8.6)	7 (11.9)	20 (8.3)
Able to carry on normal activity	90	11 (18.3)	16 (25.0)	9 (15.5)	15 (25.4)	51 (21.2)
Normal	100	44 (73.3)	44 (68.8)	41 (70.7)	35 (59.3)	164 (68.0)
Not done		0 (0.0)	1 (1.56)	1 (1.72)	0 (0.0)	2 (0.8)
Hormonal receptor status, N (%)	ER+	44 (73.3)	47 (73.4)	39 (67.2)	46 (78.0)	176 (73.0)
	PR+	38 (63.3)	43 (67.2)	34 (58.6)	42 (71.2)	157 (65.1)
Previous hormonal therapy, N (%)	Yes	44 (73.7)	48 (75.0)	40 (69.0)	46 (78.0)	178 (73.9)
	No	16 (26.7)	16 (25.0)	17 (29.3)	13 (22.0)	62 (25.7)
Visceral metastases, N (%)	Absence	19 (31.7)	19 (29.7)	16 (27.6)	23 (39.0)	77 (32.0)
	Presence	41 (68.3)	45 (70.3)	42 (72.4)	36 (61.0)	164 (68.0
Previous adjuvant/neoadjuvant taxane therapy^b^, N (%)	Yes	21 (35.0)	24 (37.5)	12 (20.7)	19 (32.2)	76 (31.5)
	No	39 (65.0)	40 (62.5)	45 (77.6)	40 (67.8)	164 (68.0)

^a^Other: 1 Creole; ^b^1 patient in Arm C had no record for previous hormonal therapy and adjuvant/neoadjuvant therapy.

Arm A: docetaxel and gemcitabine with 3-weekly schedule; Arm B: paclitaxel and gemcitabine with 3-weekly schedule; Arm C: docetaxel and gemcitabine with weekly schedule; Arm D: paclitaxel and gemcitabine with weekly schedule.

PR: Partial Response.

The median number of administered chemotherapy cycles was 6 in all arms. Treatment-related discontinuations occurred in 6.7%, 6.2%, 10.3% and 13.6% of patients in Arms A, B, C and D, respectively, while almost 50% of discontinuations were due to lack of efficacy (see also Figure 1 for details).

### Efficacy

**Figure 2:**
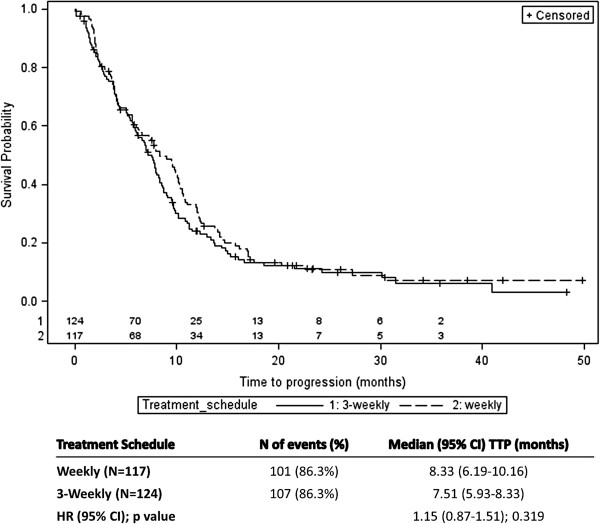
**Results of time-to-progression (TTP) by treatment schedule (Weekly vs. 3-Weekly).** Number of patients still at risk for 3-weekly (1) and weekly (2) treatment schedule are reported above the X axis.

**Figure 3:**
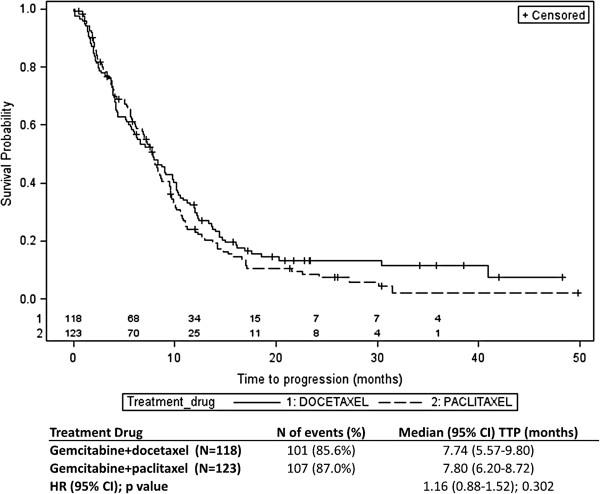
**Results of time-to-progression by treatment regimen (gemcitabine+docetaxel vs. gemcitabine+paclitaxel).** Number of patients still at risk for 3-weekly (1) and weekly (2) treatment regimen are reported above the X axis.

**Figure 4:**
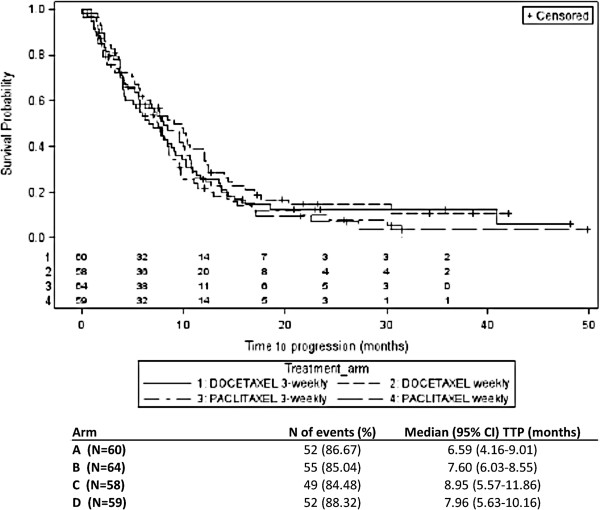
**Results of time-to-progression by treatment arm (gemcitabine+docetaxel vs. gemcitabine+paclitaxel).** Number of patients still at risk for each treatment arm (1 - Arm A: docetaxel and gemcitabine 3 weekly; 2 - Arm C: docetaxel and gemcitabine weekly 3 - Arm B: paclitaxel and gemcitabine 3 weekly; 4 - Arm D: paclitaxel and gemcitabine weekly) are reported above the X axis.

The summary results and Kaplan-Meier curves of TTP by treatment schedule and by treatment drug are shown in Figures 2 and 3, respectively. For the treatment schedule, 101 (86.3%) and 107 (86.3%) patients showed progression in the weekly and 3-weekly treatment schedules, respectively. The difference between the median TTP of the two treatment groups was not statistically significant (8.33 months (95% CI: 6.19-10.16) for the weekly schedule group versus 7.51 months (95% CI: 5.93-8.33) for the 3-weekly schedule; (HR: 1.15; 95% CI: 0.87-1.51; p-value=0.319). From the Cox regression analysis, similar results were obtained (HR: 1.14; 95% CI: 0.87-1.50; p-value=0.345) when adjusted for treatment drug and visceral metastases (the only covariate that significantly influenced TTP [p-value=0.023]). For the TTP comparison between the two treatment drugs assignment, the number of patients who showed progression was 101 (85.6%) and 107 (87.0%) for docetaxel and paclitaxel treatment groups, respectively. There was no statistically significant difference in the median TTP between the two treatment drugs group. Median TTP was 7.74 months (95% CI: 5.57-9.80) and 7.80 months (95% CI: 6.20-8.72), respectively for the docetaxel and the paclitaxel group (HR: 1.16; 95% CI: 0.88-1.52; p-value=0.302). From the Cox regression model, similar results were obtained when adjusted for treatment schedule and visceral metastases as covariates (HR: 1.23; 95% CI: 0.93-1.62; p-value=0.150). Overall, no difference in TTP was observed between the four treatment arms (Figure 4).

The median OS was 21.11 months (95% CI: 17.28-26.75) and 20.95 months (95% CI: 18.92-33.21) for the weekly schedule and the 3-weekly schedule, respectively and no statistically significant difference was observed (HR: 0.98; 95% CI: 0.69-1.37; p-value=0.886). With regard to the two doublets, the median OS was 19.11 months (95% CI: 16.59-24.0) and 23.80 months (95% CI: 19.38-31.97) for docetaxel and paclitaxel, respectively, with no statistically significant difference (HR: 1.01; 95% CI: 0.71-1.42; p-value=0.980). None of the covariates assessed using the Cox regression had a significant influence on OS.

Table 3 shows ORR results by treatment schedule and by treatment drug. A total of 241 patients were evaluable for response (117 and 121, respectively for the weekly and the 3-weekly schedule, and 117 and 121, respectively for the docetaxel and paclitaxel treatment group). In the comparison between treatment schedules, the ORR was significantly higher in the weekly treatment schedule compared to the 3-weekly treatment schedule (odds ratio; 0.504; 95% CI: 0.299-0.850; p-value=0.010) from the logistic regression model adjusted for covariates. No statistically significant differences were observed in the comparison between treatment drugs (p-value=0.639).

**Table 3:** Results of ORR by treatment schedule (Weekly vs. 3-Weekly) and by treatment drug (gemcitabine+docetaxel vs. gemcitabine+paclitaxel)

	**Treatment schedule**		**Treatment drug**	
	**Weekly**	**3-weekly**	**Gemcitabine + docetaxel**	**Gemcitabine + paclitaxel**
	**(*N*= 117)**	**(*N*= 121)**	**(*N*= 117)**	**(*N*= 121)**
	***N*(%)**	***N*(%)**	***N*(%)**	***N*(%)**
Complete response (CR)	8 (6.8)	7 (5.8)	6 (5.1)	9 (7.4)
Partial response (PR)	51 (43.6)	34 (28.1)	45 (38.5)	40 (33.1)
Stable disease (SD)	28 (23.9)	50 (41.3)	38 (32.5)	40 (33.1)
Progressive disease (PD)	25 (21.4)	16 (13.2)	21 (17.9)	20 (16.5)
Not available	1 (0.9)	2 (1.7)	1 (0.9)	2 (1.7)
Not assessed	4 (3.4)	12 (9.9)	6 (5.1)	10 (8.3)
**ORR (CR + PR)**	**59 (50.4)**	**41 (33.9)**	**51 (43.6)**	**49 (39.8)**
Odds Ratio (95% CI); p value	0.504 (0.299-0.850); 0.010		0.882 (0.523-1.488); 0.639	

CI: Confidence Interval, ORR: Overall Response Rate; unadjusted odds ratio are presented in the table.

In each treatment arm, there was a substantial reduction (i.e., >50% overall) in the number of patients who completed the QoL questionnaire at the post therapy visit when compared to those obtained at the beginning of the treatment. Therefore, the results (data not shown) are of poor reliability.

### Safety

A total of 3 patients were randomized but not treated (1 in the docetaxel 3-weekly group and 2 in the paclitaxel 3-weekly group) and were excluded from the safety population (238 patients). Overall, treatment-emergent adverse events (TEAEs) were reported in 224 patients (94.1%) while 37 patients (15.5%) experienced at least one treatment-emergent serious adverse event (TESAE). Six patients (2.5%) died due to adverse events. Chemotherapy-related TEAEs and TESAEs were reported in 218 (91.6%) and 17 (7.1%) patients, respectively. Grade 3/4 toxicities were reported in 168 (70.6%) subjects. Twenty-two (9.2%) patients discontinued the study due to TEAEs, while 14 patients (5.9%) were without TEAEs. These results stratified by treatment drugs and schedule are presented in Table 4.

**Table 4:** Summary of TEAEs and TESAEs in the four groups

	**Arm A**	**Arm B**	**Arm C**	**Arm D**
	**(*N*=59)**	**(*N*=62)**	**(*N*=58)**	**(*N*=59)**
		***N*(%)**	***N*(%)**	***N*(%)**	***N*(%)**
TEAEs:				
Subject with at least one TEAE	57 (96.6)	58 (93.5)	53 (91.4)	56 (94.9)
Subject discontinued the study due to TEAE	4 (6.8)	4 (6.5)	6 (10.3)	8 (13.6)
Subjects with at least one chemotherapy-related TEAE	56 (94.9)	57 (91.9)	50 (86.2)	55 (93.2)
Subjects without TEAE	2 (3.4)	4 (6.5)	5 (8.6)	3 (5.1)
TESAEs:				
Subject with at least one TESAE	9 (15.3)	10 (16.0)	7 (12.1)	11 (18.6)
Subjects with at least one chemotherapy-related TESAE	5 (8.5)	4 (6.5)	3 (5.2)	5 (8.5)

Arm A: docetaxel and gemcitabine with 3-weekly schedule; Arm B: paclitaxel and gemcitabine with 3-weekly schedule; Arm C: docetaxel and gemcitabine with weekly schedule; Arm D: paclitaxel and gemcitabine with weekly schedule.

TEAE: Treatment-emergent Adverse Events, TESAE: Treatment-emergent Serious Adverse Events.

Grade 3/4 toxicities occurred in 69.2% and in 71.9% of patients receiving docetaxel and paclitaxel, respectively, and in 65.8% and in 75.2%, in the weekly and 3-weekly regimen, respectively. As shown in Table 5, neutropenia (55.6% and 52.1% for docetaxel and paclitaxel group, respectively, and 45.3% and 62.0% for weekly and 3-weekly schedule, respectively) and leucopoenia (18.8% and 14.0% for docetaxel and paclitaxel group, respectively and 11.1% and 21.5% for weekly and 3-weekly schedule, respectively) were the most frequent grade 3/4 toxicities.

**Table 5:** Summary of most common grade 3 and grade 4 toxicities in the four groups (i.e., reported in ≥5% of patients in any group)

	**Arm A**		**Arm B**		**Arm C**		**Arm D**	
	**(*N*=59)**		**(*N*=62)**		**(*N*=58)**		**(*N*=59)**	
	**G3**	**G4**	**G3**	**G4**	**G3**	**G4**	**G3**	**G4**
Neutropenia	24 (40.7)	22 (37.3)	22 (35.5)	7 (11.3)	15 (25.9)	4 (6.9)	23 (39.0)	11 (18.6)
Leukopenia	15 (25.4)	1 (1.7)	7 (11.3)	3 (4.8)	6 (10.3)	0 (0.0)	6 (10.2)	1 (1.7)
ALT increased	1 (1.7)	0 (0.0)	5 (8.1)	0 (0.0)	8 (13.8)	0 (0.0)	6 (10.2)	0 (0.0)
Diarrhoea	0 (0.0)	2 (3.4)	0 (0.0)	0 (0.0)	4 (6.9)	0 (0.0)	0 (0.0)	0 (0.0)
Myalgia	0 (0.0)	0 (0.0)	4 (6.5)	0 (0.0)	0 (0.0)	0 (0.0)	0 (0.0)	0 (0.0)
Alopecia	4 (6.8)	0 (0.0)	0 (0.0)	0 (0.0)	1 (1.7)	0 (0.0)	1 (1.7)	0 (0.0)
Hepatotoxicity^a^	0 (0.0)	0 (0.0)	1 (1.6)	0 (0.0)	0 (0.0)	0 (0.0)	3 (5.1)	0 (0.0)
Asthenia	4 (6.8)	0 (0.0)	3 (4.8)	0 (0.0)	5 (8.6)	0 (0.0)	6 (10.2)	1 (1.7)
Fatigue	3 (5.1)	0 (0.0)	3 (4.8)	0 (0.0)	3 (5.2)	0 (0.0)	4 (6.8)	0 (0.0)

Data are number (%) of patient; G = CTC Grade.

^a^ALT are included in hepatotoxicities, but were separately reported by different study investigators.

Arm A: docetaxel and gemcitabine with 3-weekly schedule; Arm B: paclitaxel and gemcitabine with 3-weekly schedule; Arm C: docetaxel and gemcitabine with weekly schedule; Arm D: paclitaxel and gemcitabine with weekly schedule.

## Discussion

Both gemcitabine/docetaxel and gemcitabine/paclitaxel are active regimens for metastatic breast cancer but it was unclear which of the two combinations has the more favourable efficacy and safety profile. Furthermore, it was unclear whether a weekly schedule would prove more active and/or less toxic than the standard 3-weekly schedule [6]. In fact, the lower doses and shorter infusion times used with weekly dosing should minimize bone marrow suppression and other toxicities associated with standard 3-weekly schedules, while maintaining the dose intensity necessary for anti-tumour activity.

This study was designed to address important information on these issues choosing dosages of combined drugs based on the information available at the time the study was designed in order to maximise the effect of each drug combination (except for the weekly combination of Paclitaxel and Gemcitabine, for which a lower dose was chosen in favour of a better haematological toxicity profile). Though, the slow accrual rate in the trial prevented the completion of the planned patients’ enrolment within a reasonable time. An interim futility analysis was performed, and it was concluded that there were neither scientific nor ethical reasons to continue enrolling patients for an additional 2-3 years, and as a result, the study was terminated prematurely, before the planned sample size population was reached. Therefore, the results obtained from these analyses should be interpreted with caution.

There was no statistically significant difference in the primary study endpoint, TTP, between the treatment regimens and the treatment schedule based on the analysed data. The median TTP was 7.74 and 7.80 months for patients treated with docetaxel plus gemcitabine and paclitaxel plus gemcitabine, respectively. Median TTP for patients treated with the weekly schedule and the 3-weekly schedule was 8.33 and 7.51 months, respectively. In any case, no difference was observed between the four treatment arms. Therefore, the two doublets were similarly effective in TTP, while it was marginally, although not significantly, prolonged with the weekly regimen compared to the 3-weekly schedule.

The difference in OS between the treatment schedules was significant neither by treatment schedule nor by treatment regimen, despite being marginally prolonged with paclitaxel.

Although the study was not designed or powered to detect statistical difference in the secondary endpoints, ORR was higher in the weekly arm compared to the 3-weekly arm. However, no significant difference in ORR between the treatment regimens was observed.

A recent paper [7] reported that the addition of Gemcitabine to docetaxel failed to prove any clinically meaningful benefit, given that no significant changes in OS were detected as compared to the taxane group. Although our study was not designed to compare double agents vs single agent therapy, it is worth to note that the study from Nielsen and co-worker was not powered to detect a benefit in survival. On the other hand, the combination Gemcitabine-Docetaxel showed increased TTP as compared to docetaxel only. With more caution, Qi et al [8] in the recently published metanalysis aiming to compare double agents vs single agent therapy in MBC setting, report that the role of combination therapy in MBC setting is still unclear, although combination chemotherapy offers significant improvement in ORR and PFS.

Toxicity was acceptable and was consistent with the known safety profile of taxanes [9]. Grade 3/4 toxicities rates were similar with docetaxel and paclitaxel (69.2% and 71.9% of patients, respectively), and showed a higher trend in the 3-weekly (75.2%) than in the weekly schedule (65.8%). Neutropenia was the most common grade 3/4 toxicity, with lower rates for the weekly (45.3%) than for the 3-weekly (62.0%) schedule, and small differences between treatment regimens (55.6% and 52.1% for docetaxel and paclitaxel group, respectively).

Although caution should be used in the interpretation of these data, the use of a weekly schedule might be associated with a lower toxicity trend, with a better maintenance of dose intensity and possibly with a better tumour response compared to 3-weekly regimen, whilst no differences between treatment schedules were observed in the primary study endpoint of time to tumour progression and no differences between drug treatments were observed in safety and efficacy.

Moreover, the results of this study confirm data from various studies supporting weekly taxane dosing as an active regimen in MBC, even in heavily pretreated, refractory disease and in elderly patients or those with poor performance status [9]. It is reasonable hypothesize that results obtained with the weekly schedule might be driven by paclitaxel. In fact, it has been previously reported that weekly administration of paclitaxel has superior efficacy over the three weekly schedule coupled with different toxicity profile [6,10] while weekly docetaxel schedule proved to be at least as efficacious as tree weekly schedule [6,11]. However, a recent review has concluded that use of paclitaxel in advanced BC given in a weekly regimen gives overall survival advantages compared with the standard every three weeks regimen [12].

## Conclusions

Despite the limitation due to its premature interruption, the present study suggests that weekly administration of a taxane, particularly paclitaxel, in combination with gemcitabine is an active regimen for MBC, and might be associated with a better tumour response as compared to the 3-weekly schedule.

## Abbreviations

BC: Breast cancer; CI: Confidence interval; CR: Complete response; HER-2: Human epidermal growth factor receptor 2; HR: Hazard ratio; ITT: Intent-to-treat; IV: Intravenous; MBC: Metastatic breast cancer; NCI-CTC: National cancer institute-common toxicity criteria; ORR: Overall response rate; OS: Overall survival; PR: Partial response; QoL: Quality of life; RECIST: Response evaluation criteria in solid tumours; RSCL: Rotterdam symptom checklist; SD: Standard deviation; TEAEs: Treatment-emergent adverse events; TESAEs: Treatment-emergent serious adverse events; TTP: Time-to-progression; W: Weekly; 3W: 3-weekly

## Competing interests

This manuscript was fully sponsored by Eli Lilly Italy S.p.A. The authors declare the following competing interest: Michele De Laurentiis received honoraria for lectures and advisory boards participation from Sanofi-Aventis and for lectures from Eli-Lilly. Davide Boy, Matteo Ceccarelli and Paolo Marchi are full time employees of Eli Lilly Italy S.p.A. Gbenga Kazeem was a consultant statistician for Eli Lilly UK. Ignazia La Torre was a full time employee of Eli Lilly Italy S.p.A.

## Authors’ contribution

MV, SDP, FC are the principal investigators, have supervised the study and have contributed to study design, interpretation of data and has substantially revised the manuscript. LDM has contributed to acquisition of data, data analysis and interpretation, manuscript writing and content review. AF, MM, MDL, AD and ILT have contributed to acquisition of data, data interpretation and content review. MC has contributed to acquisition of data, data analysis and interpretation, and content review**.** GK, DFM, PB and DB have contributed to data analysis, data interpretation and content review. PM has contributed to data analysis and interpretation, manuscript writing, and content revision. All the authors approved the final version of this manuscript.

## Authors’ information

Ignazia La Torre and Gbenga Kazeem are former employee.

## Pre-publication history

The pre-publication history for this paper can be accessed here:

http://www.biomedcentral.com/1471-2407/13/164/prepub
